# Mechanical compression regulates tumor spheroid invasion into a 3D collagen matrix

**DOI:** 10.1088/1478-3975/ad3ac5

**Published:** 2024-04-15

**Authors:** Mrinal Pandey, Young Joon Suh, Minha Kim, Hannah Jane Davis, Jeffrey E Segall, Mingming Wu

**Affiliations:** 1 Department of Biological and Environmental Engineering, Cornell University, 306 Riley-Robb Hall, Ithaca, NY 14853, United States of America; 2 Department of Biological Sciences, Cornell University, 216 Stimson Hall, Ithaca, NY 14853, United States of America; 3 Department of Pathology, Albert Einstein College of Medicine, 1300 Morris Park Avenue, Bronx, NY 10461, United States of America

**Keywords:** 3D ECM, tumor compression, invasion, tumor microenvironment, tumor spheroid

## Abstract

Uncontrolled growth of tumor cells in confined spaces leads to the accumulation of compressive stress within the tumor. Although the effects of tension within 3D extracellular matrices (ECMs) on tumor growth and invasion are well established, the role of compression in tumor mechanics and invasion is largely unexplored. In this study, we modified a Transwell assay such that it provides constant compressive loads to spheroids embedded within a collagen matrix. We used microscopic imaging to follow the single cell dynamics of the cells within the spheroids, as well as invasion into the 3D ECMs. Our experimental results showed that malignant breast tumor (MDA-MB-231) and non-tumorigenic epithelial (MCF10A) spheroids responded differently to a constant compression. Cells within the malignant spheroids became more motile within the spheroids and invaded more into the ECM under compression; whereas cells within non-tumorigenic MCF10A spheroids became less motile within the spheroids and did not display apparent detachment from the spheroids under compression. These findings suggest that compression may play differential roles in healthy and pathogenic epithelial tissues and highlight the importance of tumor mechanics and invasion.

## 1. Introduction

Solid tumor stress is a key indicator of tumor physiology. Clinically, the feel, touch, and shape of a solid tumor are important diagnostic methods for malignancy of the tumor [1–3]. In advanced tumors, rapid tumor cell growth in a confined tissue environment often leads to the build up of solid stresses. The resistance of the surrounding host tissue for making room to the growing tumor, causes the compressive stresses within the tumor core [4, 5]. Compressive stresses can press on the blood or lymphatic vessels within the tumor [5, 6] leading to heightened interstitial fluid pressure [7, 8]. The tension around the tumor periphery, an important component of the solid stress, is a well-studied biomarker for tumor malignancy. Cell traction forces re-organize and align collagen matrices surrounding the tumor and invasion into interstitial space [9–11]. Taken together, mechanistic understanding of roles of solid tumor stress in tumor invasion is important for potential therapeutical intervention for the treatment of solid tumors.

The mechanical environment of tumor cells can critically regulate tumor invasion. Landmark work from the Weaver lab has shown that tension within the tumor microenvironment promotes tumor progression [3]. Recently, work from our labs and others has shown that breast tumor cells and the cancer associated fibroblasts align, stiffen, and plastically deform the surrounding extracellular matrix (ECM), and the stiffened ECM in return promotes cell force generation and invasion [12, 13]. Although how tension within the tumor microenvironment regulates tumor progression is extensively investigated, there is limited work on how compressive stress influences tumor mechanics and invasion [14]. This is in part due to the lack of tools that can provide controlled compressive stress for tumors in a physiologically realistic setting and at the same time compatible with optical microscopy.

Studies of roles of compression on tumor cell migration have mostly focused on single cells. In a 2D setting where cells were plated on a substrate, compression can enhance the migration and invasion of breast [15–17], pancreatic [18] and brain cancer cells [19]. There is limited work in a more physiologically realistic 3D setting where cells were embedded within 3D ECMs [20, 21]. In native states, tumors are compressed in a confined space typically surrounded by ECMs. Previous work in our labs has shown that tumor spheroids are more sensitive to biochemical cues compared to single cells [22].

To further the compression studies to a more physiologically realistic setting, we modified a 3D Transwell assay to investigate the effect of compression on normal and breast tumor spheroids embedded within collagen. The Transwell assay is compatible with single cell imaging within as well as outside the tumor spheroid. We find differential responses from malignant breast tumor spheroids (MDA-MB-231) and non-tumorigenic epithelial spheroids (MCF10A) when compressed, with MDAMB-231 cells becoming more motile and invasive while MCF10A cells becoming less motile in response to compression.

## 2. Materials and Methods

### 2.1. Cells, spheroids, and 3D spheroid culture preparation

#### Cells.

Metastatic breast adenocarcinoma cells (MDA-MB-231 cell line) and non-tumorigenic epithelial cells (MCF10A cell line) were provided by the Cornell Center of Microenvironment and Metastasis. MDAMB-231 were cultured for up to 20 passages, and were used at 50%–70% confluency [23]. The growth medium for MDA-MB-231 cells was composed of DMEM high glucose medium (Catalog No. [Cat.] 11965092, Gibco, Life Technologies Corporation, Grand Island, NY), 10% fetal bovine serum (Cat. S11150, Atlanta Biologicals, Lawrenceville, GA), and 1% antibiotics (100 units ml^*−*1^ penicillin and 100 *μ*g ml^*−*1^ streptomycin, Cat. 15140122, Gibco). MCF-10A cells were cultured up to passage 10 and used at 70%–90% confluency. The growth medium for MCF-10A cells was composed of DMEM/F-12 medium (Cat. 11320033, Gibco), 5% donor horse serum (Cat. S12150, Atlanta Biologicals), 20 ng ml^*−*1^ human EGF (Cat. PHG0311, Gibco), 0.5 *μ*g ml^*−*1^ hydrocortisone (Cat. H0888–1G, SigmaAldrich, St. Louis, MO), 100 ng ml^*−*1^ Cholera Toxin (resuspend at 1 mg ml^*−*1^ in sterile DI H2O, Cat. C8052-.5MG, Sigma-Aldrich), 10 *μ*g ml^*−*1^ insulin (Cat. 10 516–5ML, Sigma-Aldrich), and 5% antibiotics (Gibco). We note that DMEM/F12 media was used for the preparation of both MDA-MB-231 and MCF10A spheroids. MDA-MB-231 expressing EGFP and MCF-10A cells expressing GFP were kind gifts from Dr Joseph Aslan at the Oregon Health & Science University. Fluorescently labeled MDA-MB-231 and MCF-10A cells were cultured in the same way as the non-labeled cells and were used for the co-culture spheroid experiments.

#### Spheroids.

Uniformly sized spheroids were generated using a specially designed microwell array platform (see figure S1) [12, 22, 24]. Briefly, a silicon master of 18 × 18 microwell array was fabricated in the Cornell Nanofabrication Facility (CNF) using a one-layer photo-lithography method. Then the microwells were patterned on a 1 mm thick and 1 × 1 cm size agarose gel using a soft lithography method×(see figure S1). Each microwell is cylindrical in shape with a diameter of 400 *μ*m and depth of 400 *μ*m. The agarose gel surface provides low adhesion of cells, thus promoting clustering of cells which results in spheroid formation. Six individual microwell arrays ~1 cm in size were then placed in 6 wells of a 12- well plate (Cat. #: 07-200-82, Corning). Within each well of the 12-well plate, 3 million cells (1:20 ratio of fluorescently labeled: non-labeled cells) suspended in 2.5 ml of DMEM/F12 growth medium were introduced. The plate was then gently placed in 5% CO_2_ incubator at 100% humidity for four days. On day 3, DMEM/F12 spheroid growth medium was replenished. We note that the architecture of MDA-MB-231 spheroids is different from MCF10A spheroids. MCF10A spheroids are very compact and are formed overnight while MDA-MB-231 spheroids are less compact and take 3–5 d to form uniform sized spheroids. We harvested the spheroids on day 5 for both the cell lines. Tumor spheroids were collected from two arrays of microwells for each experiment and filtered by a Falcon Cell Strainer (Cat. #: 352360, Corning) with 100 *μ*m pores to ensure the uniformity of the spheroid size. It is important to note that rich media and 5 d culture is important to form uniform sized MDA-MB-231 tumor spheroids.

#### Spheroid embedded ECM.

To make 3D tumor spheroid cultures, we suspended spheroids in a 3.5 mg ml^*−*1^ type I collagen matrix (rat tail tendon Cat. #: 354249, Corning). Briefly, for each experiment, 200 *μ*l of spheroid embedded collagen mixture was prepared with a collagen concentration of 3.5 mg ml^*−*1^. To do this, 73.68 *μ*l of collagen stock (9.5 mg ml^*−*1^) was first titrated with 1.62 *μ*l 1N NaOH and 20 *μ*l 10X M199 (Cat. #: M0650–100Ml, Sigma) to yield a final pH of *~*7.4 [25]. Then, 104.69 *μ*l of spheroids with DMEM or DMEM/F12 GM for MDA-MB231 and MCF10A spheroids respectively was added to reach a final volume of 200 *μ*l. On average there were 1620 spheroids per ml of collagen. The final average spheroid concentration was approximately 4–5 spheroids per *in vitro* device (about 1 spheroid per mm^2^ under the top view).

### 2.2. *In vitro* device setup

**Figure 1.**
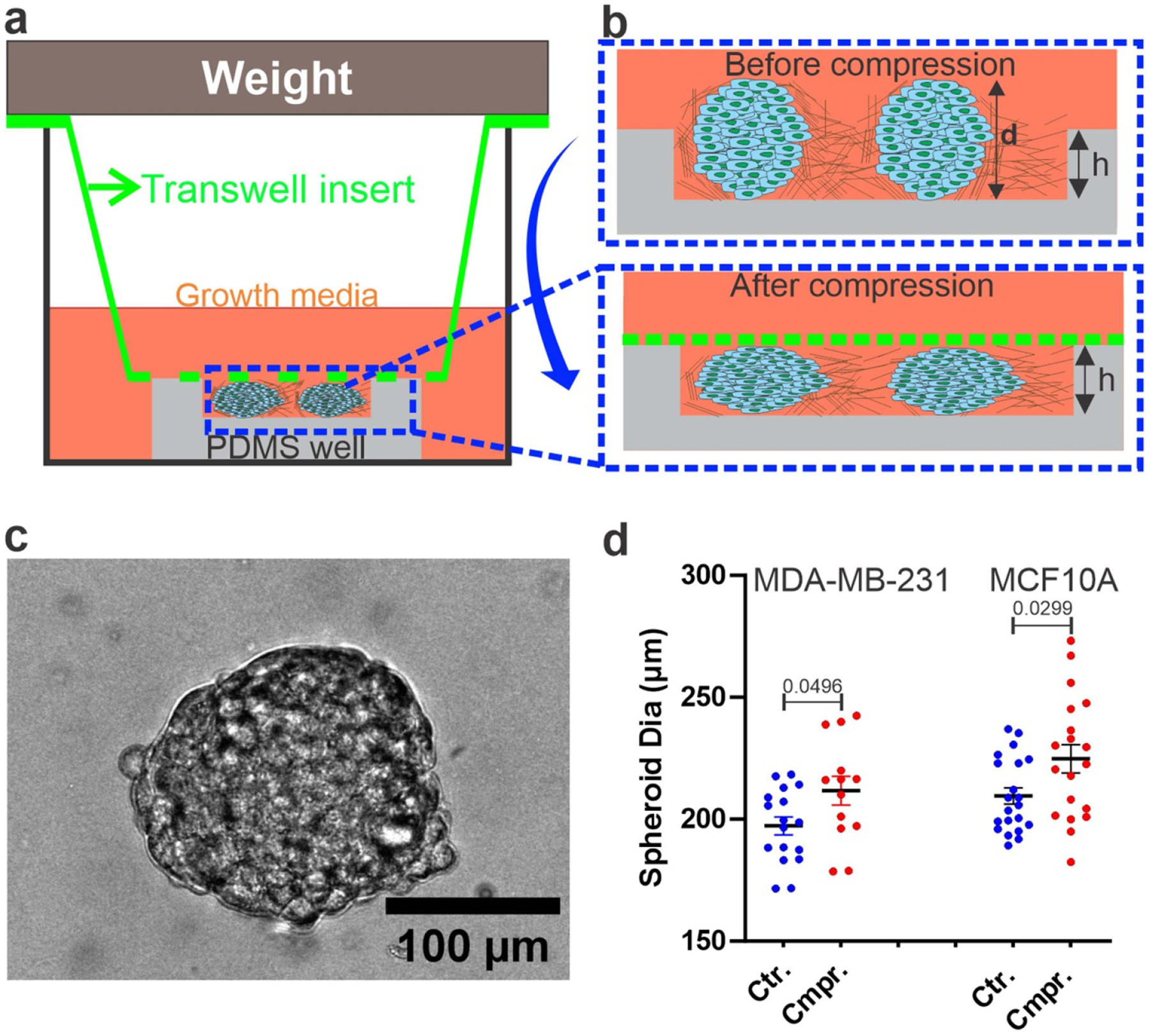
An *in vitro* model for mechanical compression of tumor spheroids: (a) cross-sectional view of a modified transwell assay for compressing tumor spheroids. Spheroid embedded collagen matrices were seeded within a PDMS well of defined depth, *h* = 115 *μ*m. A weight was placed on top of the transwell insert for compressing the tumor spheroids. The bottom of the transwell insert is a porous Polystyrene membrane with pore size of 8 *μ*m. Cell culture media were placed both in the 24 well plate and the bottom of the transwell to a height of 3 mm above the PDMS well. (b) A zoom in view of the cross-section of spheroid embedded collagen before and after compression. Before compression, the spheroid diameter (*d*) is 197.27 ±3 *μ*m for MDA-MB-231 and 209.53 ± 3 *μ*m for MCF10A spheroids. After compression the spheroid height is same as the well depth h, the compression strain rate is computed as (*d − h*)/*d* which is *~*40%. Note when the transwell insert was used to compress the spheroid, we made sure that the polystyrene membrane made direct contact with top of the PDMS well. (c) Brightfield image of an MDA-MB-231 spheroid surrounded by 3.5 mg ml^*−*1^ collagen, taken at *t* = 0, where *t* = 0 is the time imaging started. (d) Scatter plot of MDA-MB-231 and MCF10A tumor spheroid diameters in the horizontal plane before (Ctr.) and after compression (Cmpr.). Each dot is data from one spheroid. The *p* values were obtained using a parametric Welch’s *t*-test compared to the control group.

A silicon master with 7 cylindrically shaped pistons of 4 mm diameter and 115 *μ*m depth was fabricated in the CNF using one-layer photolithography. The 1 mm thick PDMS membrane with 7 wells were patterned from the silicon master using soft lithography and was placed at the base of one of the wells in a 24-well plate. The 1 mm thickness of the PDMS membrane is critical to ensure that Transwell insert sits perfectly on top of the PDMS well. Spheroid-embedded collagen was introduced in the PDMS well. A Transwell insert was placed on top of the PDMS well after the collagen was polymerized (see figure 1). A static load of 35 g (metallic ring) was then placed on top of the Transwell insert to compress the spheroids. The 8 *μ*m pore size Transwell insert were chosen to ensure sufficient exchange of media and nutrients. For sterility, PDMS wells were autoclaved and both the PDMS wells and the 24-well plate (Cat #: 353047) were treated with oxygen plasma (Harrick Plasma Cleaner PDC-001, Harrick Plasma, Ithaca, NY) for 1 min on high power mode.

#### Surface activation.

To ensure optimal surface properties for the binding of the collagen, the surface of the PDMS well was activated using 1% poly(ethyleneimine) (Cat. P3143–100ML, Sigma-Aldrich) for 10 min followed by thorough rinsing, using sterilized DI water, and treating the device with 0.1% Glutaraldehyde (Cat. 16019, Electron Microscopy Sciences, Hatfeld, PA) for 30 min. The PDMS wells were again thoroughly rinsed three times. The PDMS wells were filled with sterilized DI water and left in the biohood overnight at room temperature.

#### 3D spheroid seeding.

On the day of an experiment, the PDMS wells-bonded 24 well-plate was placed on an ice pack for the entire device setting up time. After allowing the PDMS wells to cool, 2.5 *μ*l of spheroid-embedded collagen solution was added to each PDMS well. The well-plate was then placed on a larger petri dish padded with wet tissues, and the petri dish was then placed in the incubator to allow collagen polymerization at 37 C and 5% CO_2_ for 30 min. We note that the temperature ramping rate during polymerization plays an important role in collagen structure [26]; to get a small and homogenous pore size network we used fast warming in our setup. To achieve fast warming the petri dish was placed on a metallic block (which was at 37 °C) instead of directly placing the petri dish in the incubator. Slow warming results in an inhomogeneous large pore size network [12]. Following the polymerization, 1 ml of DMEM or DMEM/F12 growth medium was added for MDAMB-231 or MCF10A experiment setup respectively. The well plate was then transferred to the microscope stage enclosed by an environmental control chamber (WeatherStation, PrecisionControl LLC), which was kept at 37 °C, 5% CO_2_ and about 70% humidity. Here *t* = 0 is the time at which the imaging started which is approximately 2 h after the spheroid embedded collagen was polymerized. Each experiment had 2 compressed conditions where a load was applied using Transwell insert (Cat #: 353097, Corning) and 2–3 control wells where no static load was applied on the spheroids. Each experiment was repeated 3 times.

### 2.3. Imaging and data analysis

All images were taken using an inverted epifluorescent microscope (IX81, Olympus America, Center Valley, PA, USA) with a CCD camera (ORCAR2, Hamamatsu Photonics, Bridgewater, NJ, USA). In all the experiments the middle z-plane of the spheroids was captured using a 20X objective (Olympus, NA = 1) in bright field and in green fluorescence. The light source for fluorescence imaging was provided by the X-Cite series 120PC unit (Excelitas Technologies, Waltham, MA, USA). The scope has a stage incubator (Precision Plastics Inc., Beltsville, MD, USA) that maintained a temperature of 37 °C, humidity of *~*70%, and 5% CO_2_ level. The setup was placed on the automated X-Y microscope stage (MS-2000, Applied Scientific Instrumentation, Eugene, OR), and images were taken every 10 min for 16 h using CellSens software (Olympus America, Center Valley, PA, USA). For each experiment brightfield and GFP images were taken.

Using the brightfield time-lapse images, the circularity of spheroids was calculated by manually drawing the outlines of the spheroids and then applying the circularity measurement parameter in ImageJ. Circularity is defined as (4*π* Area/perimeter Perimeter^2^) [27]. The cell trajectories were obtained using the manual tracker in ImageJ (National Institute of Health) using the GFP time-lapse images. Single cell migration parameters of speed, persistence length and mean squared displacement (MSD) were calculated using these trajectories [23]. The cell speed is defined as the total length of the track divided by the time duration. The cell persistence length is defined as the distance between the cell starting and ending positions divided by the length of the cell trajectory. Parametric *t*-test (Welch test) was carried out using Prism (GraphPad Software, Inc., La Jolla, CA). To quantify the protrusion over time we define a new metric called Roughness Index. To calculate the roughness index, we fit an ellipse on the spheroid. The ratio of perimeter of spheroids to the circumference of the ellipse gives the measure of roughness of spheroid. The rationale for this concept is that when there are no protrusions, the spheroid will be a smooth ellipse. With protrusions the perimeter of the spheroid will increase thus increasing the roughness. A higher value of roughness index would result in more jaggedness or protrusions of spheroids.

## 3. Results and discussion

### 3.1. A modified Transwell assay to apply static mechanical compression on tumor spheroids compatible with microscopic imaging at single-cell resolution

A commercial Transwell assay was modified to apply well-defined mechanical compression to spheroids embedded within a 3D collagen gel. The key modification to the Transwell assay was the addition of a 1 mm thick PDMS membrane patterned with a microwell (4 mm diameter and 115 *μ*m depth) and placed at the bottom of a 24-well plate (figure 1(a)). The depth of the PDMS well, *h*, is critical because it determines the amount of compressive strain that can be applied to the spheroid. The compressive strain was defined as (*d − h*)/*d*, where d is the diameter of the spheroid. For each experiment, PDMS membranes with the microwell were first fabricated using a standard soft lithography technique and adhered to the bottom of a 24-well plate (figures 1(a) and (b)). Spheroid-embedded collagen was then introduced into each of the PDMS wells, and the collagen was allowed to polymerize. Subsequently, the spheroids were compressed by placing a Transwell insert with a metallic weight on top. The bottom of the insert was ensured to be in direct contact with the top of the PDMS membrane (figure 1(b)). We note that the bottom of the Transwell insert is made of a 10 *μ*m thick, porous polyester PET membrane with average pore size of 8 *μ*m and the diameter of the insert is 6.5 mm. The PET membrane allowed continuous nutrient and gas exchange between the 3D spheroid culture and the surroundings for the duration of the experiments (16 h). In a typical experiment, we used four out of the 24 wells, and the spheroids were observed using an automatic translational stage on the microscope.

To create spheroids of a targeted size, a previously developed microwell array device was used (see figure S1 and also [24].) The diameters of the spheroids were calculated using bright-field microscopic images, as shown in figure 1(c). The size of the spheroids depended on the initial cell seeding density, total number of incubation days, and size of the microwell. Our hydrogel-based array microwell device provides a robust way to create spheroids of well-defined sizes. In this study, the average diameters in the horizontal plane of the generated MDA-MB-231 tumor spheroids were 197.27 ± 3 *μ*m in the control (uncom-pressed) and 211.68 ± 6 *μ*m in the compressed condition (figure 1(d)). Similarly, the average diameter of MCF10A spheroids was 209.53 ± 3 *μ*m in the control and 224.78 ± 5 *μ*m in the compressed condition (figure 1(d)). The average compressive strain was determined as the difference between the average spheroid diameter (197.27 *μ*m) and the height of the PDMS well (115 *μ*m) divided by the spheroid diameter, which was 41% for the MDA-MB-231 spheroids. Similarly, the average compressive strain for the MCF10A spheroids was 44%.

The unique capability of our modified Transwell assay is its compatibility with microscopic imaging, allowing us to follow single-cell dynamics within spheroids. In addition, it provides a straightforward method to apply compression to spheroids. Although only one compressive strain rate was used in our study, the compressive strain rate can be changed by using PDMS wells of different sizes. For MDA-MB-231 and MCF10A spheroids the variation of spheroid diameter within the group was ±15.1 *μ*m. (see figure 1(d)), which leads to a strain rate variation of 0.097 within the group. The disadvantage of our setup is that it does not allow for dynamic compression. To overcome this limitation, a microfluidic rheometer is currently being developed in our lab.

### 3.2. Compression differentially regulates the motility of metastatic MDA-MB-231 and normal epithelial MCF10A cells within the spheroids

Tumor mechanics has been a key indicator of tumor malignancy [28, 29]. To explore spheroid mechanics, we followed single-cell motility within spheroids.

#### 3.2.1. Compression enhanced motility of MDA-MB-231 cells but suppressed that of MCF-10A cells within spheroids

**Figure 2.**
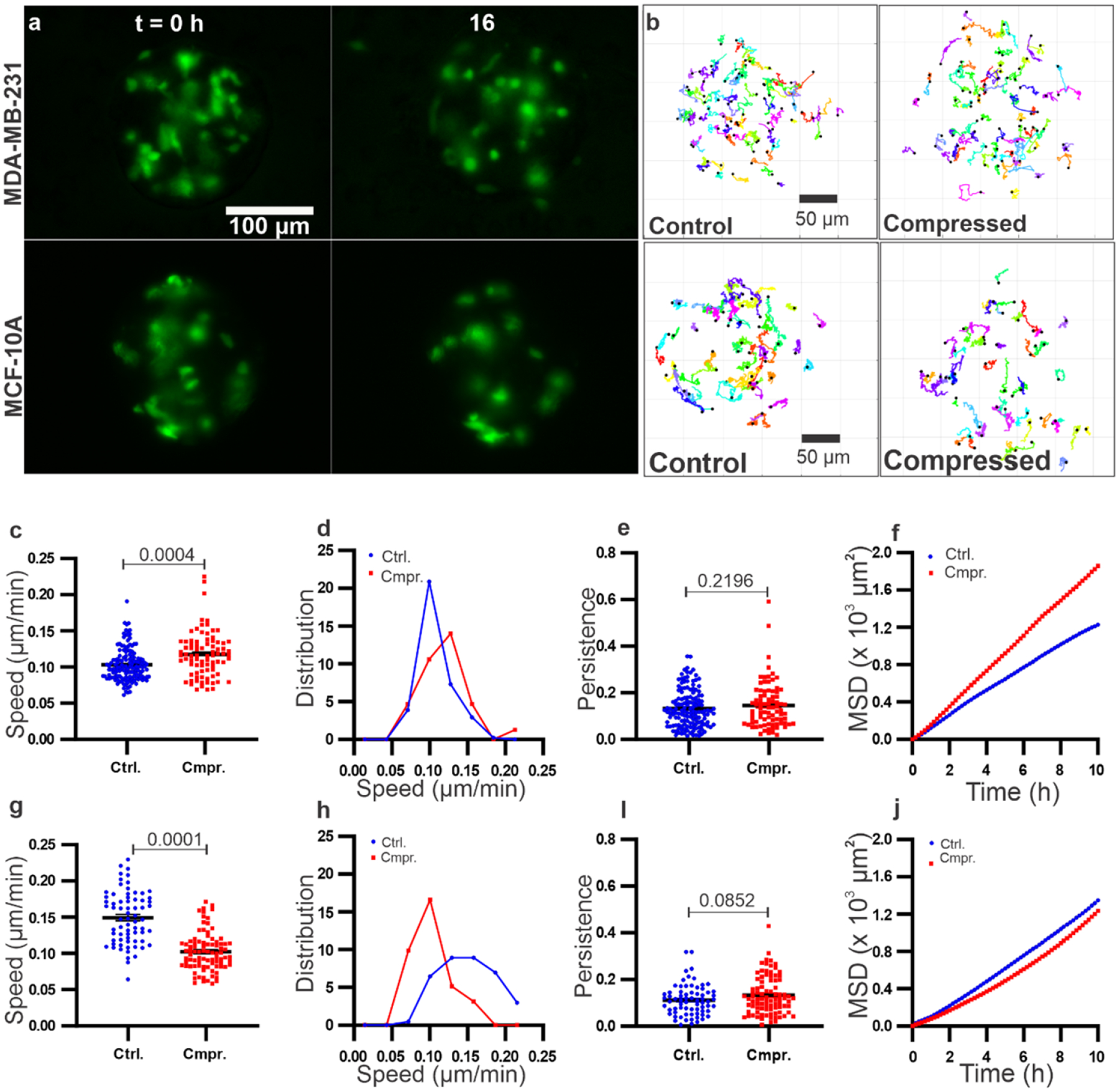
Characterization of single-cell dynamics within tumor spheroids under control and compressed conditions. (a) Fluorescence images of MDA-MB-231 and MCF10A tumor spheroids taken at *t* = 0 and *t* = 16 h. A mixture of 1:20 fluorescent:non-fluorescent cells was used to make spheroids to facilitate single cell imaging within the spheroid. (b) Trajectories of MDA-MB-231 and MCF10A cells within the tumor spheroids in control and compressed conditions. Each colored line is a cell trajectory of cells within the spheroids. Each cell trajectory shown here is 10 h long. A total of 80 cells were randomly tracked using 17 and 13 spheroids for control and compressed conditions for MDA-MB-231 and a total of 70 cells were tracked using 21 and 19 spheroids for control and compressed conditions for MCF10A spheroids, respectively. (c)–(f) Cell migration speed (c), distribution of speed (d), persistence length (e), MSDs (f) of cells within MDA-MB-231 spheroids in control and compressed conditions. (g)–(j) Cell migration speed (g), distribution of speed (h), persistence length (i), MSDs (j) of cells within MCF10A spheroids in control and compressed conditions. The data here was computed using tracks shown in panel (b). The *p* values were obtained using Welch’s parametric *t*-test compared to the control group.

To study spheroid mechanics under compression, we investigated the motility of cells within tumor spheroids. To follow cell movement within the spheroid, we used a mix of 1:20 GFP labeled tumor cells to non GFP labeled tumor cells (figure 2(a)). Sixteen-hour long time series of images of the fluorescent cells (see figure 2(a) and movies S3–S6) were obtained using an epi-fluorescence microscope. Each single-cell trajectory within the spheroid was tracked using the manual tracker in ImageJ, along with an in-house developed MATLAB code (figure 2(b)).

To quantify the motility of cells within the spheroids, we computed the cell motility parameters using the trajectories shown in figure 2(b). Specifically, we obtained the speed, persistence length (plength), and MSD of the cells within the spheroid under compression and in the control (see figures 2(c)–(j)). Within MDA-MB-231 spheroids, the cell speed was significantly enhanced by compression at an average speed of 0.117 ± 0.003 *μ*m min^*−*1^, in contrast to the control at 0.103 ± 0.001 *μ*m min^*−*1^ (figures 2(c) and (d)). There was no significant change in persistence length (figure 2(e)). One way to evaluate tumor spheroid mechanics is to use the diffusion coefficient, assuming that the cells are executing a random walk. To examine how cells diffuse within the spheroids, we computed the MSDs and found that MSDs were greater in compressed in contrast to control in MDA-MB-231 spheroids (figure 2(f)). Using the first-order approximation for MSD, where MSD = 4Dt, we have the diffusion coefficient *D* = 0.047 ± 0.0003 *μ*m^2^ min^*−*1^ for the compressed condition in contrast to *D* = 0.031 ± 0.0005 *μ*m^2^ min^*−*1^ for the control. Similarly, we computed the motility parameters for MCF10A spheroids. Surprisingly, compression had the opposite effect on the MCF10A spheroids. The average speed of cells in compressed MCF10A spheroid condition was lower (0.102 ± 0.002 *μ*m min^*−*1^) than that of the control (0.154 ± 0.004 *μ*m min^*−*1^) (figures 2(g) and (h)). The persistence length for MCF10A spheroids in the compressed state was 0.129 ± 0.009, in contrast to the control at 0.108 ± 0.009 (figure 2(i)), with no statistical significance observed. On examining the MSDs of the MCF10A cells within spheroids, we found that the MSD was significantly reduced upon compression, in contrast to the control (figure 2(j)). We computed the diffusion coefficient *D* = 0.030 ± 0.001 *μ*m^2^ min^*−*1^ for the compressed condition, in contrast to *D* = 0.033 ± 0.0007 *μ*m^2^ min^*−*1^ for the control. It is also interesting to note that MCF10A cells move faster than MDA-MB-231 cells within the spheroids, their MSD curves (see figures 2(f) and (j)) show that MCF10A are more superdiffusive than MDA-MB-231 cells. We also observed a subtle time-dependent relationship in both type of cells. For MDA-MB-231 cells we found no major difference in speed of cells in the compressed and control conditions for first 8 however, after that the speed of cells in compressed condition significantly increased in comparison to control (Welch’s *t* test, *p* value < 0.0001, see figure S2(a)). Whereas in MCF10A spheroids, the speed of cells in the compressed and control conditions were different from *t* = 1 h (see figure S2(b)).

Taken together, the speed and MSDs measurements suggest that cells within MDA-MB-231 tumor spheroids became more motile, whereas cells within MCF10A spheroids became less motile upon compression. These changes were result of speed change, not persistence length change. While the reason for this differential response requires further investigation, the work presented here is consistent with previous work carried out on the compression of confluent 2D cell sheets [15, 16]. In these studies, a wound healing assay was used to quantify the motility of cells. It was found that motility of malignant cells, including MDA-MB-231 and 4T1 cells, was enhanced when compressed, while motility of non-malignant MCF10A cells was suppressed upon compression. We also noted that in our compression study non-malignant MCF10A cells migrated faster than malignant MDA-MB-231 cells within the spheroids. Similar behavior has been reported previously, where MCF10A cells migrated faster than MDA-MB231 cells when they were plated on a 2D substrate or embedded within a 3D collagen gel [15, 30].

### 3.3. Compression differentially regulates invasion of MDA-MB-231 cells and MCF10A cells into the collagen matrix

Tumor cell invasion is an important step during cancer metastasis [23, 31]. Here, we asked whether compression regulates tumor invasion into 3D ECMs.

#### 3.3.1. Compression decreases the circularity of MDA-MB-231 spheroids but does not have observable effects on MCF10A spheroids

**Figure 3.**
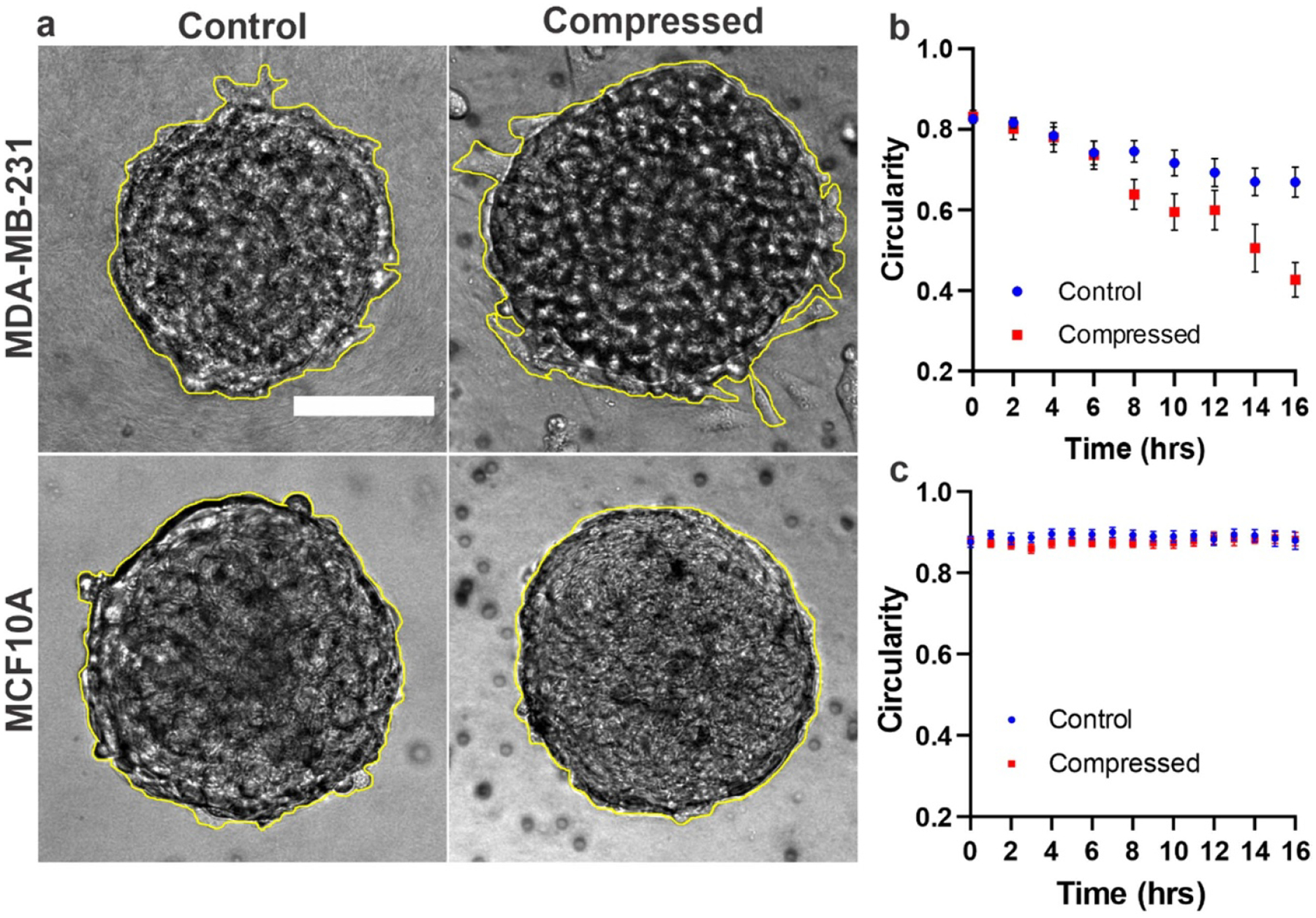
Compression regulates morphology of MDA-MB-231 spheroids but has no apparent effect on MCF10A spheroid morphology. (a) Brightfield images of MDA-MB-231 and MCF10A spheroids embedded in 3.5 mg ml^*−*1^ collagen in control and compressed condition at *t* = 16 h. The center of spheroid in *z* direction was selected as the focus plane for imaging. The scale bar is 100 *μ*m (b) Change in circularity of MDA-MB-231 spheroids over a period of 16 h in control and compressed conditions. (c) Change in circularity of MCF10A spheroids over a period of 16 h in control and compressed conditions. In (b) and (c), the spheroid outlines were generated manually and then the circularity of spheroids were calculated using ImageJ. Data in figure (b) is from three separate experiments. A total of 17 spheroids were used in control and 13 in compressed condition. Data in figure (c) are from three separate experiments. A total of 21 spheroids were used in control and 19 in compressed condition.

**Figure 4.**
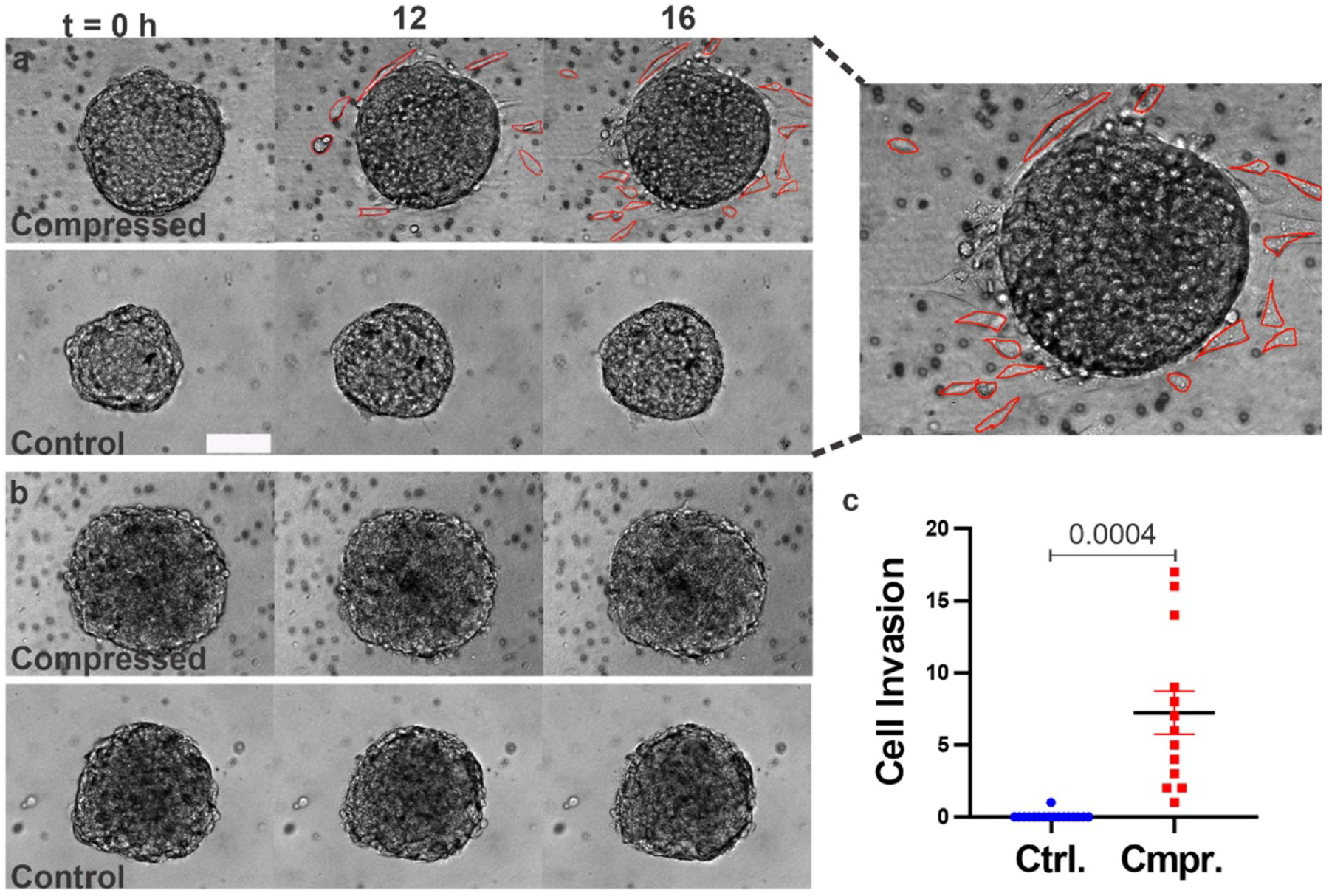
Compression promoted invasion of MDA-MB-231 cells into the ECM and had no observable effect on MCF10A spheroids. (a) Time-lapse images of MDA-MB-231 spheroids in control and compressed conditions. On the right is the zoomed in image of a MDA-MB-231 tumor spheroid at *t* = 16 h. Cells that invaded into the ECM are outlined with bright red lines. The imaging plane is the center of the spheroid. The scale bar is 100 *μ*m. (b) Time-lapse images of MCF10A spheroids in control and compressed conditions. (c) Cells that broke away from the spheroid and invaded into the ECM were manually counted using ImageJ for MDA-MB-231 spheroids. A parametric Welch’s *t*-test compared to the control group.

Time-lapse brightfield images of spheroids embedded in ECM were recorded with and without compression (figure 3(a)). The circularity parameter, defined as the ratio of the 4 * pi area to the perimeter squared, was used to quantify the spheroid shape. Note that a circularity value of 1 (maximum) indicates that the spheroid is perfectly circular, and a decreasing value from 1 reflects a deviation from circularity. The outlines of the spheroids were traced manually, as shown by the bright yellow lines in figure 4(a), and the parameters were calculated using ImageJ. The time evolution of the circularity at 2 h intervals in MDA-MB231 and MCF10A spheroids is shown in figures 3(b) and (c), respectively. In MDA-MB-231 spheroids, compression led to a significant change from circular shape to a less circular shape over time. The circularity values changed from 0.832 to 0.427 under compression and from 0.831 to 0.669 under the control condition (figure 3(b)). In contrast, MCF10A spheroids maintained a close-to-circular shape at all time points regardless of compression or control conditions. The circularity values ranged from 0.8995 to 0.859 under both control and compressed conditions (figure 3(c)). The observed significant decrease in circularity of MDA-MB-231 spheroids occurred after 6 h of compression, suggesting a time-dependent response to compression.

Here we used circularity as a measure of tumor spheroid peripheral roughness. A sphere corresponds to a geometry where the surface volume ratio is the smallest; hence, the amount of least roughness at the spheroid ECM interface. We found that MDA-MB-231 spheroids became rougher over time under compression compared to the control. Upon careful examination of the images, we found that the roughness was due to protrusions around the spheroids, a first step before cells detached from the spheroids. In parallel, the MCF10A spheroids showed no clear response to compression. Thus, in addition to circularity we also looked at roughness index of the spheroids (see figure S3 and methods section for details). We found that roughness of MDA-MB-231 spheroids in compressed condition significantly increased after 6 h which is consistent with circularity change observation. We note that the roughness of tumor periphery has been used as a diagnostic tool for tumor malignant tumors in previous literature for melanoma [32].

#### 3.3.2. Compression promoted the invasion of MDA-MB-231 cells into the ECM but not MCF10A cells

Cells that broke away from the spheroids and invaded into the ECM were manually counted using time-lapse bright-field images (see figure 4 and movie S1). The bright red lines highlight cells that invaded into the ECM. We found that on average, 7 ± 2 MDA-MB-231 cells per spheroid broke away from compressed tumor spheroids during the 16 h observation time in contrast to average no cells breaking away per spheroid in control (see figure 4(c)). We also looked at the distribution of cells invading into collagen matrix in MDA-MB-231 spheroids with time (see figure S4), we found that invasion rate of cells is not uniform with time rather it increases with time. Similarly, we investigated the invasion of MCF10A cells into the ECM, but we did not observe any cells invading into the ECM under either compressed or control conditions during 16 h of experiment (figure 4(b) and movie S2). Given that we used 1:20 (GFP labeled:non labeled) cells, we used brightfield images to count the cells that invaded the ECM independent of the GFP label to ensure comprehensive recording of cells that invaded the ECM. We note that the imaging focus plane is the center of the spheroids which is determined by moving the objective lens up and down in z direction (see figure S5).

Taken together, both circularity and single-cell invasion studies demonstrated that compression promoted MDA-MB-231 tumor cell invasion but had no apparent impact on MCF10A cell invasion. In addition, we observed time dependency in both the spheroid circularity and single-cell breakout experiments. In MDA-MB-231 spheroids, we observed significant invasion only after the first 6–8 h (see movie S1), and this was observed quantitatively in both circularity and single-cell invasion. MCF10A cells may take longer to invade into the ECM than the 16 h that we followed them for; thus, longer experiments would provide more information on the invasion patterns of MCF10A spheroids.

## 4. Conclusion and future perspectives

In this study, we developed a 3D *in vitro* tumor spheroid model to quantitatively investigate the effects of compression on tumor spheroid mechanics and invasion. Our results showed that compression had differential effects on the mechanics and invasion of malignant and non-tumorigenic epithelial spheroids. Upon compression, malignant MDA-MB-231 cells became more motile within the spheroids and more invasive into the ECM, whereas in non-tumorigenic MCF10A spheroids, motility within spheroids was suppressed, and no invasion was observed.

Differential responses to mechanical compression of malignant and non-tumorigenic cells can be caused by the mechanosensing ion channel, Piezo1. It has been reported that malignant cells have substantially higher levels of Piezo1 than non-tumorigenic MCF10A cells [33, 34]. Previous studies have also shown that Piezo1 activates integrin [35–37]. Considering the higher levels of Piezo1 in malignant MDA-MB-231 cells, compared to non-tumorigenic MCF10A cells, it makes sense that MDA-MB-231 cells respond to compression more sensitively than that of MCF10A. Additionally, MDA-MB231 cells have higher level of integrins than MCF10A [38], which further contributes to the observed differential response. Overall, our results are consistent with what was reported in 2D wound healing assays, in that malignant cancer cells are more mechanosensitive and compression selectively stimulates malignant tumor cell invasion compared to normal cells [39].

The next challenge is to fully understand the molecular mechanisms underlying the differential responses of malignant and non-tumorigenic spheroids to compression. It is important to first identify the key molecular players using a general approach, including RNA sequencing, to gain an unbiased insight into all transcripts. Systematic studies on breast cancer cells with different invasive potentials, including MCF7, T47D can also reveal and validate the key molecular players. It would be interesting to conduct studies on other tumor types, such as brain tumors, in which the malignant state of the tumors is often highly compressed. Systematic investigation of the effects of compression on different tumors can potentially answer the question whether mechanosensitivity is correlated with tumor malignancy. Here, our work established a straightforward *in vitro* model to investigate the effects of compression on tumor spheroids, allowed for studies of their subsequent invasion in a 3D microenvironment, and highlighted the importance of compression in tumor mechanics and invasion. The limitation of our model is that it can only provide a static compression. *In vivo* compression typically happens in a dynamic way. Our group is currently developing a microfluidic device for dynamic compression of the tumor spheroids, a platform compatible with optical imaging. In addition, this platform will enable us to change strain rate easily. We anticipate that dynamic compression experiments will provide a more complete understanding of the relation of tumor mechanics and invasion.

## Supplementary Material

S1.mp4

S2.mp4

S3.mp4

S4.mp4

S5.mp4

S6.mp4

SupplementaryMaterials.pdf

Supplementary material for this article is available online

## Data Availability

The data cannot be made publicly available upon publication because no suitable repository exists for hosting data in this field of study. The data that support the findings of this study are available upon reasonable request.
