# Supplementary material for: Mechanical compression regulates tumor spheroid invasion into a 3D collagen matrix
Source: Phys Biol. Author manuscript; Available in PMC 2026 Mar 1. (PMC12950161; doi:10.1088/1478-3975/ad3ac5)
Supplement: SupplementaryMaterials.pdf [file NIHMS2147544-supplement-SupplementaryMaterials_pdf.pdf]

Mechanical compression regulates tumor spheroid invasion into a 3D collagen matrix.

Mrinal Pandey[1], Young Joon Suh[1], Minha Kim[2], Hannah Jane Davis[2], Jeffrey E Segall[3], and Mingming Wu[1]

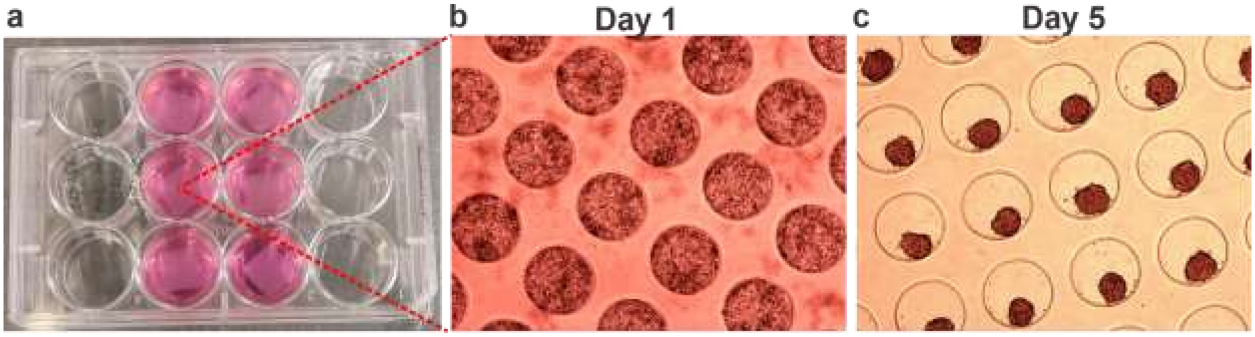

Figure S1: High throughput tumor spheroid formation assay. A) Image of a 12 well plate containing high throughput agarose based microwell array for spheroid formation. Each microwell device has 18x18 microwells patterned agarose gel membrane, and each microwell is 400 µm in diameter and depth. In a typical experiment 2 of these arrays are used to harvest spheroids. **B)** Micrograph of tumor spheroid formation in a microwell array. A total of 3 million MDA-MB-231 cells are seeded on day 1 to each microwell array. **C)** The prepared spheroids are harvested on day 5 and the media is changed regularly every 2-3 days. MCF10A spheroids were made in the same device.

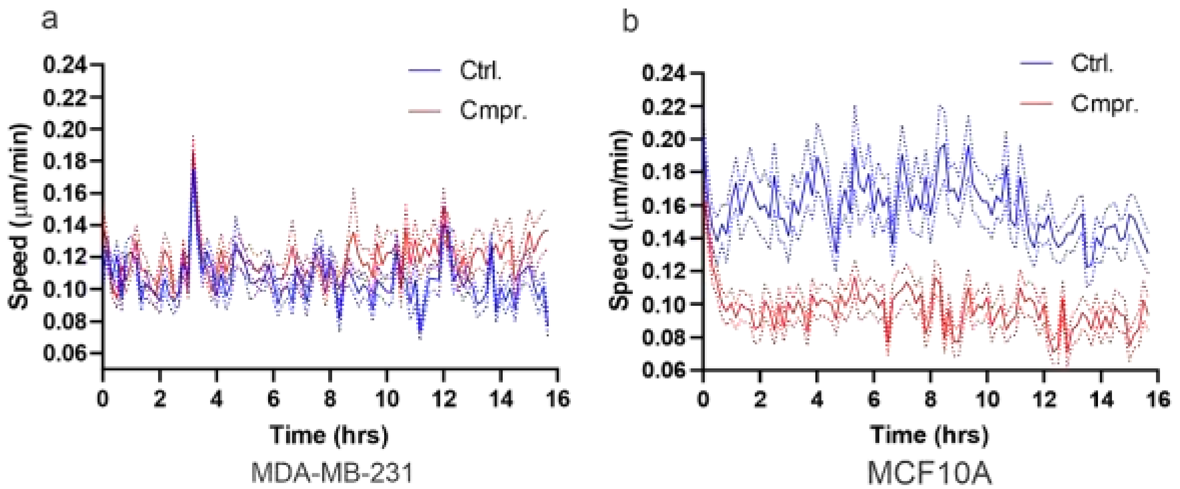

Figure S2: Time evolution of speed in MDA-MB-231 and MCF10A spheroids. A) Speed of MDA-MB-231 cells within spheroids **B)** Speed of MCF10A cells within spheroids as a function of time under compressed and control conditions.

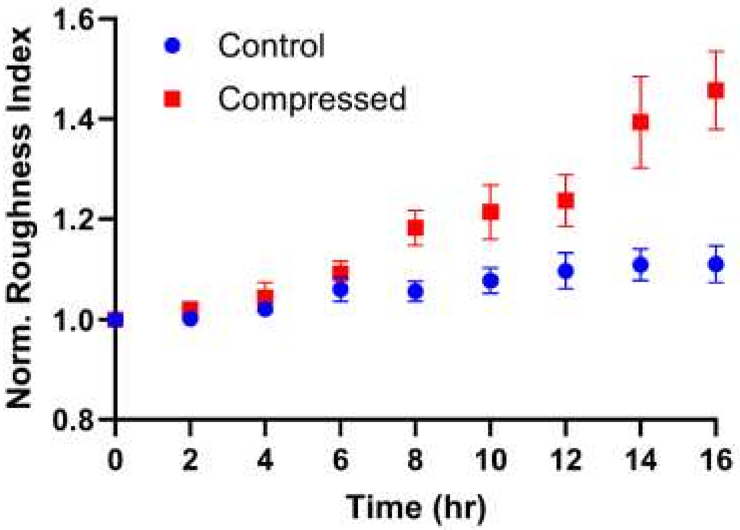

Figure S3: Compression regulates the roughness of tumor spheroids. Using the manually generated spheroids outlines, roughness index of MDA-MB-231 tumor spheroids was calculated over a period of 16 hours in control and compressed conditions. A total of 17 spheroids were used in control and 13 in compressed condition.

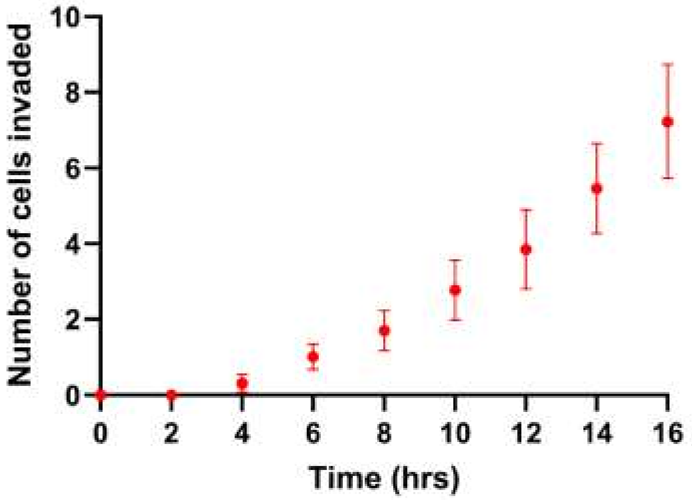

Figure S4: Evolution of single cell invasion into the ECM with time. The average number of MDA-MB-231 single cells that invaded into the ECM over a period of 16 hours.

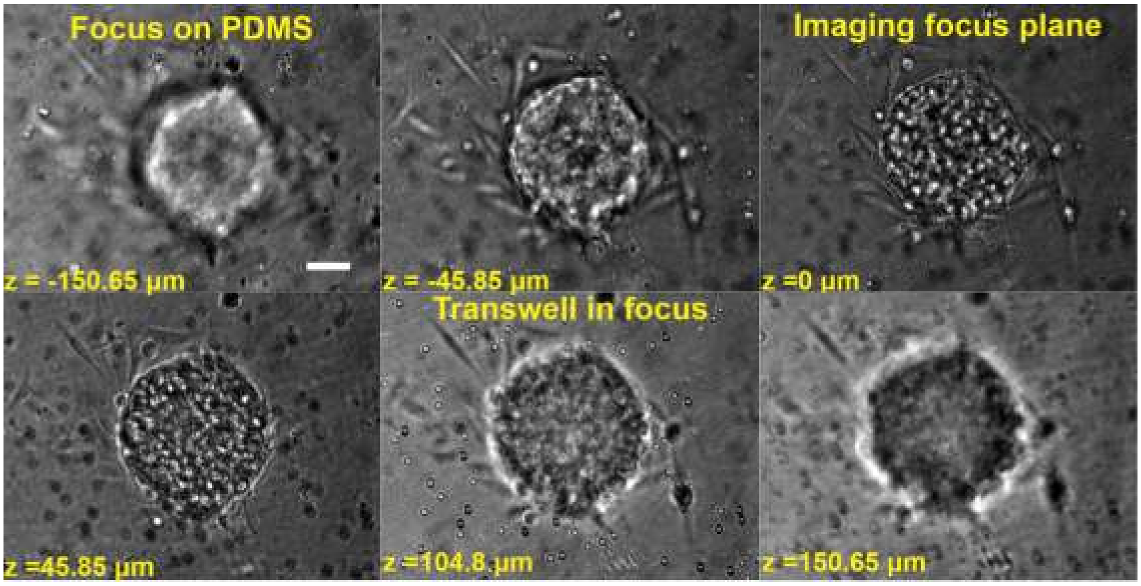

Figure S5: Bright field images of MDA-MB-231 tumor spheroid taken in z direction at t= 16 hour. Positive z is above the midplane, and negative is below. Scale bar is 50 µm.

Movies:

All the movies below were taken of spheroids embedded within a 3.5 mg/mL collagen. Each image is 430 μm × 330 μm, the time between consecutive image is 10 minutes, and the duration of the move is 16 hours. The frame rate of movies is 16 fps.

Movie S1: **MDA-MB-231 tumor spheroids embedded in ECM in control and compressed condition.** Concatenated bright field video of MDA-MB-231 tumor spheroids embedded in 3.5 mg/ml collagen in control and compressed condition.

Movie S2: **MCF10A spheroids embedded in ECM in control and compressed condition.** Concatenated bright field video of MCF10A spheroids embedded in 3.5 mg/ml collagen in control and compressed condition.

Movie S3: **Motion of MDA-MB-231 cells within a spheroid with no compression.** Video of MDA-MB231 spheroids taken by a fluorescence microscope. The spheroid consists of 1:20 ratio of fluorescently labeled and nonlabelled MDA-MB-231 cells under compression.

Movie S4: **Motion of MDA-MB-231 cells within a spheroid under compression.** Video of MDA-MB231 spheroids taken by a fluorescence microscope. The spheroid consists of 1:20 ratio of fluorescently labeled and nonlabelled MDA-MB-231 cells under compression.

Movie S5: **Motion of MCF10A cells within a spheroid with no compression.** Video of MCF10A spheroids taken by a fluorescence microscope. The spheroid consists of 1:20 ratio of fluorescently labeled and nonlabelled MDA-MB-231 cells under compression.

Movie S6: **Motion of MCF10A cells within a spheroid under compression.** Video of MCF10A spheroids taken by a fluorescence microscope. The spheroid consists of 1:20 ratio of fluorescently labeled and nonlabelled MDA-MB-231 cells under compression.
